# Methadone-induced encephalopathy: a case series and literature review

**DOI:** 10.1186/s12880-020-0410-9

**Published:** 2020-01-17

**Authors:** Maryam Haghighi-Morad, Zahra Naseri, Nazila Jamshidi, Hossein Hassanian-Moghaddam, Nasim Zamani, Leila Ahmad-Molaei

**Affiliations:** 1 Department of Radiology, Loghman-Hakim Hospital, Shahid Beheshti University of Medical Sciences, Tehran, Iran; 2 Drug Health Services, Sydney Local Health District, Sydney, NSW Australia; 3 Social Determinants of Health Research Center, Shahid Beheshti University of Medical Sciences, Tehran, Iran; 4 Department of Clinical Toxicology, Loghman Hakim Hospital, Shahid Beheshti University of Medical Sciences, Tehran, Iran; 5 Neuroscience Research Center, Shahid Beheshti University of Medical Sciences, Tehran, Iran

**Keywords:** Methadone, Acute encephalopathy, Delayed leukoencephalopathy, Magnetic resonance imaging

## Abstract

**Background:**

Accidental ingestion or consumption of supra-therapeutic doses of methadone can result in neurological sequelae in humans. We aimed to determine the neurological deficits of methadone-poisoned patients admitted to a referral poisoning hospital using brain magnetic resonance (MR) and diffusion weighted (DW) imaging.

**Methods:**

In this retrospective study, brain MRIs of the patients admitted to our referral center due to methadone intoxication were reviewed. Methadone intoxication was confirmed based on history, congruent clinical presentation, and confirmatory urine analysis. Each patient had an MRI with Echo planar T1, T2, FLAIR, and DWI and apparent deficient coefficient (ADC) sequences without contrast media. Abnormalities were recorded and categorized based on their anatomic location and sequence.

**Results:**

Ten patients with abnormal MRI findings were identified. Eight had acute- and two had delayed-onset encephalopathy. Imaging findings included bilateral confluent or patchy T2 and FLAIR high signal intensity in cerebral white matter, cerebellar involvement, and bilateral occipito-parietal cortex diffusion restriction in DWI. Internal capsule involvement was identified in two patients while abnormality in globus pallidus and head of caudate nuclei were reported in another. Bilateral cerebral symmetrical confluent white matter signal abnormality with sparing of subcortical U-fibers on T2 and FLAIR sequences were observed in both patients with delayed-onset encephalopathy.

**Conclusions:**

Acute- and delayed-onset encephalopathies are two rare adverse events detected in methadone-intoxicated patients. Brain MRI findings can be helpful in detection of methadone-induced encephalopathy.

## Background

Methadone is a synthetic opioid that is increasingly used as an analgesic and in maintenance therapy of opioid-addicted patients [1, 2]. Accidental ingestion of methadone or consumption of its supra-therapeutic doses have been shown to cause multi-organ damage in both humans and animals [1, 3–5].

There have been several previous case reports describing acute-onset encephalopathy (AOE) and delayed-onset leukoencephalopathy (DOL) as adverse complications of methadone intoxication [4–6]. AOE presents with MRI abnormalities within the first admission of the patient. DOL, however, manifests with abnormalities detected on MRI in patients who have initially responded to treatment (complete resolution of symptoms), but are then re-admitted after a period of lucidity (usually days to weeks post the primary event) with neurological or psychiatric deterioration [7–10].

AOE is one of the severe neurologic complications of methadone intoxication, that has previously been associated with carbon monoxide and heroin toxicities [7, 8]. To date, eight case reports have been published reporting AOE associated with methadone toxicity, ages ranging from 22-month-old to 65 years old [1, 4, 5, 11–16]. These cases have reported a range of neurologic complications, including restrictive diffusion throughout the cerebral gray matter, bilateral diffuse cerebellar edema or infarction, hippocampal and basal ganglia (globus pallidus), fluid-attenuated inversion recovery (FLAIR) intensities, absence of central intracranial blood flow, supra and infratentorial gray matter thickening, and non-enhancing T2 hyperintensities and restriction diffusion in the white matter of both hemispheres with sparing of subcortical U fibers [4, 5, 11–16].

DOL was first described in a 24-year-old patient who developed apathy and disorientation after the initial improvement from a mixed methadone-benzodiazepine poisoning [16]. Other case studies have reported a range of DOL symptoms, including disorientation, paranoid and bizarre behavior, and severe progressive cognitive decline with bilateral cerebral white matter hyperintensities. MRI changes in these case reports have included diffuse abnormal T2 and FLAIR signals in the corona radiate, centrum semiovale and subcortical white matter throughout all lobes, and signal abnormalities in temporomesial, substantia nigra, and basal ganglia [6, 8, 9, 12, 13, 17–21]. The aim of our study was to identify and describe the pattern of neurological deficits and associated brain magnetic resonance imaging (MRI) changes in methadone-poisoned patients.

## Methods

In this retrospective file audit, the clinical records of all patients admitted to our referral poisoning hospital with the diagnosis of methadone intoxication between May 2016 and March 2018 were reviewed. A total of 2930 cases were identified, of whom only 10 fulfilled the inclusion criteria.

### Definitions

Methadone intoxication was defined based on history, clinical presentations of respiratory depression (opioid toxidrome) or loss of consciousness (LOC) responsive to administration of naloxone, as well as detection of methadone in urine analysis. The patients were classified into two subtypes: acute- and delayed-onset encephalopathy (AOE and DOL, respectively) based on clinical history. Patients with persistent neurological deficits in their first admission were categorized to have AOE based on their MRI changes. Those who had been discharged after either complete or partial recovery from acute intoxication, but then deteriorated with neurological signs or symptoms within several days or weeks necessitating readmission were considered to have DOL [7–9]. The most prevalent delayed symptoms included psychotic delirium, fluctuating state of consciousness, depression, apathy, and bizarre behaviors [9–13]. Complete knowledge of time courses and clinical presentation was a prerequisite in categorization of the patients. Imaging was performed due to persistent neurological deficits several days after admission or if there was re-occurrence of neurotoxicity after a lucid interval of at least 1 week.

### Inclusion criteria

AOE: Patients who had been admitted due to methadone intoxication and had undergone imaging due to persistent neurological deficits were enrolled in AOE group.

DOL: Neurological deficits were defined as a deterioration of neurologic function leading to readmission within one to 3 weeks after discharge without any new toxic exposure. Patients fulfilling this criteria were enrolled into the DOL group.

### Exclusion criteria

If methadone diagnosis was not confirmed after the review of the history, presentation, and urine analysis. Patients who had co-ingestions confirmed by urine analysis were also excluded (e.g. Alcohol). Any cases with possible intoxication, with a coingestants known to cause MRI complications (carbon monoxide [CO], methanol, cyanide, etc.) were excluded.

### Imaging

Scans were performed by a 1.5-T multi-planar MRI device. Echo planar T1 (TR: 591 ms, TE:15 ms, Spatial Resolution: 6.2 mm slice thickness, FoV: 230 mm*230 mm), T2 (TR: 4048 ms, TE:90 ms, Spatial Resolution: FLAIR, diffusion weighted imaging (DWI) and apparent deficient coefficient (ADC) sequences without contrast media were performed. The scan time was 15 min. All images were reviewed by a single radiologist experienced in MRI. Detected abnormalities were recorded and categorized based on their anatomic location and sequence. The areas with both restriction in DWI and low signal in abnormal diffusion restriction (ADC) were considered abnormal.

## Results

Eight patients had a brain MRI performed during their first admission due to persistent neurological deficit despite active treatment (AOE group; Tables 1 and 2). This group included four children (aged 23 months to 16 years) who had accidentally ingested methadone. The other two had developed new neurological deficits days after the initial recovery from intoxication (DOL group; Table 3). All ten patients had abnormal findings on MRI.

**Table 1:** Clinical characteristics of patients with AOE

Patient number	Age/ Sex	Initial presentation	Urine toxicology	Time to imaging
1	13 yr, F	Cyanosis	Methadone	Day 5
2	16 yr, F	Apnea	Methadone	Day 7
3	23mo, M	Cyanosis	Methadone, benzodiazepine	Day 5
4	30 yr, M	Intoxication then witnessed apnea	Methadone	Day 2
5	31 yr, M	Confusion then witnessed apnea	Methadone, opiate	Day 12
6	32 yr, F	LOC	Methadone	Day 3
7	33 yr, M	LOC	Methadone, opiate	Day 2
8	5 yr, M	LOC	Methadone	Day 2

**Table 2:** Brain MRI findings in patients with AOE

Number	Age/ Sex	Bilateral cerebral white matter T2 and FLAIR hyperintensity	Bilateral cerebellar white and gray matter T2 and FLAIR hyperintensity	Bilateral parieto-occipital T2 and FLAIR hyperintensity (PRES features)	Internal capsule involvement	Other structures	Infarction	Hemorrhage
1	13 yr, F	Yes	Yes	Yes	–	–	No	No
2	16 yr, F	Yes	–	–	–	–	No	No
3	23mo, M	Yes	–	–	–	–	No	No
4	30 yr, M	–	Yes	Yes With restriction in DWI and low signal in ADC sequences	–	–	No	No
5	31 yr, M	Yes	–	–	Yes	Splenium of corpus callosum	No	No
6	32 yr, F	–	–	Yes With restriction in DWI and low signal in ADC sequences	–	–	No	No
7	33 yr, M	Yes	–	–	Yes with restriction in DWI and ADC sequences	–	No	No
8	5 yr, M	–	Yes	Yes With restriction in DWI and low signal in ADC sequences	–	Globus pallidus and caudate nuclei	No	No

**Table 3:** Clinical characteristics and brain MRI findings of patients with DOL

Number	Age/ Sex	Toxic agent	Clinical presentation in relapse phase	Time to relapse after initial intoxication	Initial imaging	Delayed phase brain MRI findings	DWI and ADC
9	47 yr, M	Methadone	Disorientation, seizure like activity	9 days	No	Confluent bilateral symmetrical cerebral white matter T2 and FLAIR hyperintensity with sparing of sub cortical U-fibers	No restriction
10	49 yr, M	Methadone	Confusion	18 days	No	Confluent bilateral symmetrical cerebral white matter T2 and FLAIR hyperintensity with sparing of sub cortical U-fibers	No restriction

### AOE group

**Fig. 1:**
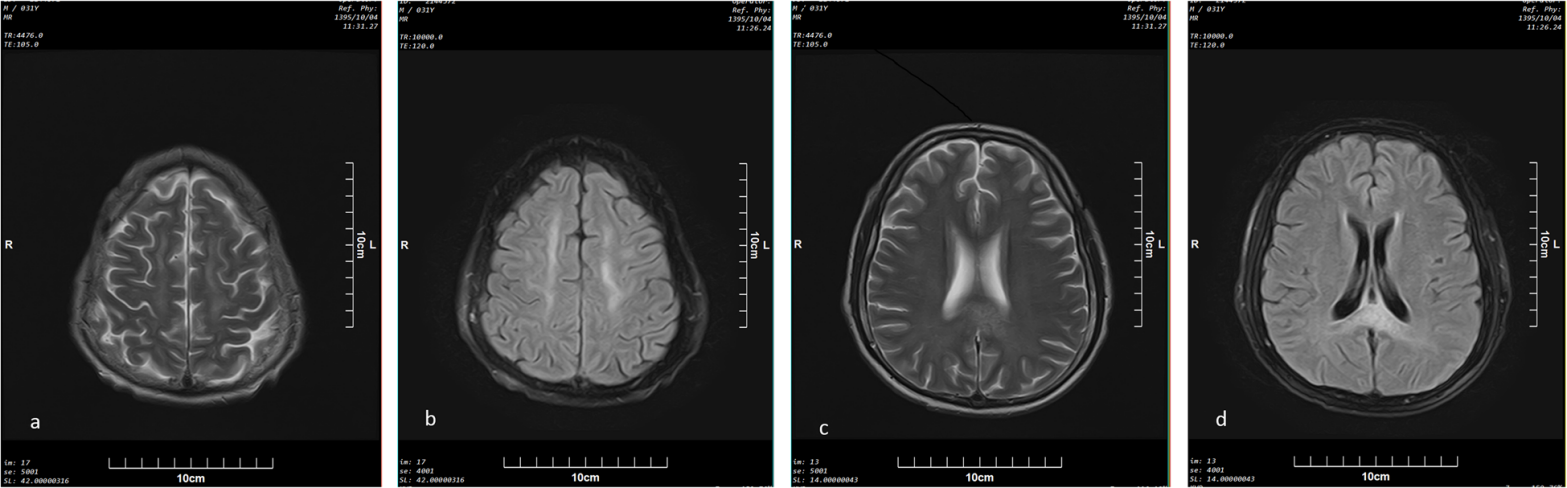
Axial (**a**) T2 and (**b**) FLAIR sequences of a 31-year-old man (patient number 5) , 12 days after presenting to emergency department with AOE symptoms, symmetric areas of hyperintensity in both centrum semiovale are seen. Axial (**c**) and (**d**) FLAIR sequences of the same patient at the mid ventricular level show hyperintensity in the splenium of corpus callosum

**Fig. 2:**
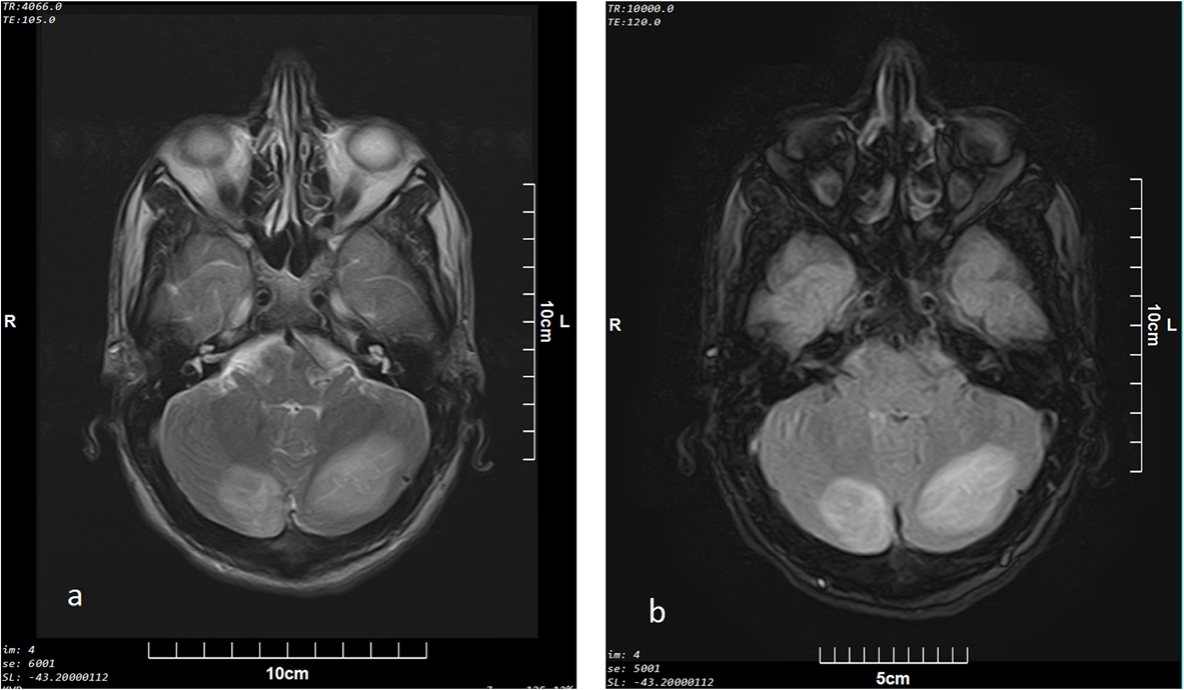
Axial (**a**) T2 and (**b**) FLAIR sequences from posterior fossa at the level of fourth ventricle in a 30 year old male ( patient number 4) two days after primary symptoms of AOE, high signal areas are seen in posterior part of cerebellum with gray and white mater involvement

**Fig. 3:**
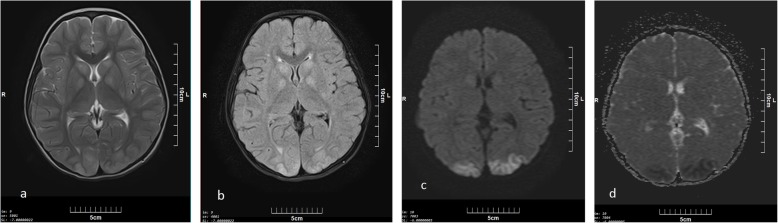
Axial (**a**) T2 and (**b**) FLAIR sequences of a 5- year-old boy (patient number 8) , two days after initial presentation of AOE , bilateral symmetric hyperintense areas are seen in basal ganglia (globus pallidus and head of caudate) in keeping with cortical and subcortical hyperintense areas in occipital lobes which has restricted in DWI (**c**) with low ADC signal (**d**)

Seven patients in this group were male and median age was 23 years [range; 23 months to 33 years). Based on urine analysis, three cases had positive urine for other drugs. One had received benzodiazepine as a part of medical management. Other two were multi-opioid abusers, but had only overdosed on methadone. Imaging findings in this group included bilateral confluent or patchy T2 and FLAIR high signal intensity areas in cerebral white matter in six (Fig. 1), cerebellar involvement in four (Fig. 2), and bilateral occipitoparietal cortex signal abnormality (low on T1 and high on T2) associated with diffusion restriction (confirmed with low signal intensity in ADC) in three cases (Fig. 3) and without restriction in one case. Internal capsule involvement was detected in two patients with hyper-signal corpus callosum in one. Abnormalities in the globus pallidus and the head of caudate nuclei were reported in only one patient. The MRIs were performed at 2- and 12-day intervals after initial presentation with methadone intoxication (defined as the primary toxic event).

### DOL group

**Fig. 4:**
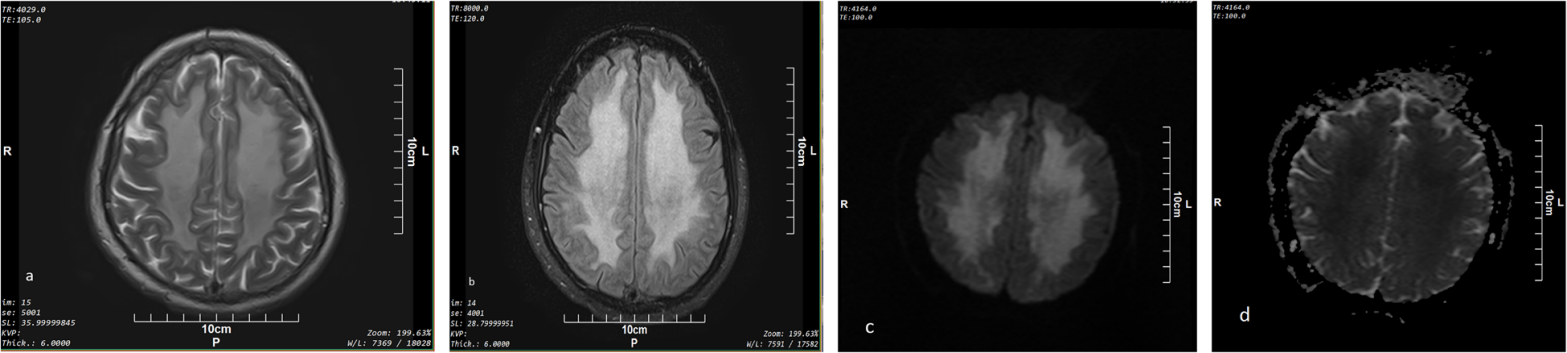
Axial (**a**) T2 and (**b**) FLAIR sequences in a 47-year-old male with DOE (patient number 9) above the ventricular level show confluent bilateral symmetrical cerebral white mater hyperintensity with sparing of subcortical U-fibers. Axial (**c**) DWI and (**d**) ADC sequences also show T2 shine through phenomenon without any evidence of diffusion restriction

The two patients in this group were 47 and 49 years old and had a 9- and 18-day lucid interval, respectively, between the initial presentation and clinical relapse (Table 3). Confluent bilateral symmetrical cerebral white matter signal abnormality with sparing of subcortical U-fibers on T2 and FLAIR sequences were observed in both of these patients (Fig. 4).

## Discussion

Methadone-induced encephalopathy is a rare event. To date, this phenomenon remains poorly characterized [4, 5, 21]. The brain MRI changes reported in the literature are summarized in Table 4 and include: cerebellum abnormalities [1, 4, 10–14], bilateral cerebral white matter abnormalities [4, 5, 11, 12, 14], signal changes in hippocampus [10], globus pallidus [13], and in a single case report in the head of caudate nuclei [4]. In addition, there is a single case report of a 2-year-old infant found to have cerebral white matter, cerebellar, and globus pallidus hypodensities based on computed tomography (CT) scan [22].

**Table 4:** Summary of published case reports of AOE

Author(s)	Age/sex	Clinical presentation	Clinical findings in discharge	Lab Data	Time to imaging	MRI findings
Anselmo M. Et al (2005) [10]	3yo, M	LOC, irregular breathing, low BP	Mild Ataxia	Urine toxicology: positive for methadone after 36 h	Day 6	High T2 in cerebellar hemispheres and hippocampus
Mills F. Et al (2008) [11]	3yo, F	LOC, hard breathing, hypothermia	Spastic dysplasia and dystonia	Urine toxicology: positive for methadone	Day 2	FLAIR: damage to gray matter and white matter of cerebellum with marked swelling
Riascos R (2008) [12]	22mo,-	LOC	Brain death	Urine toxicology: positive for methadone, acetaminophen and salicylate	In admission day	Diffuse bilateral cerebellar infarction, absence of central intra cranial blood flow, supra and infra tentorial gray matter thickening
Corré J. Et al (2013) [13]	29yo, M	LOC, hypothermia, bradypnea	Good recovery, except persistent renal failure and kinetic cerebellar syndrome	Blood analysis was positive for alcohol, cannabis, methadone (146 ng/ml), and benzodiazepines	At first day of admission.	FLAIR and DWI: high in both cerebellar and basal ganglia (globus pallidus)
Metkees M. Et al (2015) [14]	15yo, F	LOC, hypothermia, hypercapnia, HTN	Death	Detailed history revealed methadone ingestion of unknown quantity	Not mentioned	T2 and DWI: high in white matter of both hemisphere (sparing sub cortical U-fibers and deep gray matter, cortical or cerebellar)
Cerase A. Et al (2011) [4]	49yo, M	LOC	Complete recovery after 3 mo.	Serum toxicology: positive for methadone	Not mentioned	T2 and FLAIR: high in white matter of right cerebellum and deep gray and white matter of both cerebral hemispheres.
R.A. Salgado et al. (2009) [5]	65yo, F	Apathy, a catatonic state with extreme rigidity, reflexes in the upper limbs, and a bilaterally positive Babinski sign	In the following month, the patient slowly recovered.	Serum and urine toxicology shows large amount of methadone	Day 27	FLAIR and T2: symmetric signal intensity abnormality in the deep white matter of both cerebral hemisphere with sparing of sub cortical U-fibers without corresponding diffusion restriction
Rando J. (2016) [1]	14yo, M	Hypothermia, HTN, respiratory depression	Aphasia, truncal ataxia	Serum toxicology: positive for methadone	In admission day	FLAIR: cereberallitis

In our AOE patients, the most frequent MRI finding was bilateral confluent or patchy cerebral white matter hyperintensity (*n* = 5). Cerebellar abnormalities were detected in only three cases despite this was the most common observed abnormality in previous studies [1, 10, 13]. A consistent (*n* = 4) and new finding in these patients was bilateral parieto-occipital cortex T2 and FLAIR hyperintensity. This radiological finding has also been reported in patients with posterior reversible encephalopathy syndrome (PRES [23];). PRES has been reported as a consequence of or in conjunction with a variety of critical illness states including severe hypertension, hemolytic-uremic syndrome, thrombocytopenic thrombotic purpura, and in association with drug toxicities such as cisplatin, cyclophosphamide, interferon [23–25], and opiates such as morphine [26, 27]. In keeping with the findings in PRES, three of our patients had bilateral parieto-occipital cortex restriction in DWI which was confirmed by ADC sequence. Additionally, restriction was observed in one patient with internal capsule involvement (case 7). Restriction in bilateral cerebral white matter has previously been reported secondary to methadone toxicity [4, 11]. One study suggested that “deep watershed infarct” resulted in the restriction imaging observed [11]. Given our observations and previous published reports, it can be postulated that the changes in AOE due to methadone could result in PRES.

We also had two patients who had internal capsule involvement. This finding is in accordance with previously published reports as a characteristic of heroin toxicity [28]. In our both patients, morphine and methadone were detected in urine analysis. Therefore, heroin use cannot be ruled out. Additional confirmatory testing for supplementary heroin metabolites would have been useful in these two individuals. However this was not available in our center. One of them (Case 5) demonstrated lesions in splenium of corpus callosum, a finding never reported before in either heroin or methadone intoxication. This finding may be a transient lesion of splenium and has been associated with various clinical conditions such as seizures, metabolic disturbances, infections, CNS malignancy, and drugs and toxins (antidepressants, antiepileptics, antipsychotics, chemotherapy agents, and pesticides) [15, 28–38]. We also had a single patient (case 8) who showed involvement of the globus pallidus and head of caudate nuclei. This finding has been observed in association with methadone toxicity [4, 13]. Previously, brain imaging changes associated with methadone intoxication were suggested to be as a consequence of hypoxic events secondary to overdose [12]. However, hypoxia-associated cerebral adverse effects on imaging seem to be only a result of prolonged hypoxia [39, 40]. Majority of our patients did not have a persistent documented hypoxic insult. Brain neuroimaging was performed on admission, and before the worsening of patient’s condition. Secondly, brain and cerebellar damage demonstrated at both diagnosis and follow-up showed a clear-cut prominent involvement of the subcortical white matter. In adulthood, hypoxic-ischemic insults usually result in watershed zone infarcts when mild to moderate, and affect the gray matter in the basal ganglia, thalami, cerebral cortex, cerebellum, and hippocampi when severe. Furthermore, severe insult generally includes a stage of diffuse cerebral edema with loss of differentiation between gray and white matter, a finding that was not noted in the patients reported. Furthermore, acute and early subacute phases of hypoxia-induced encephalopathy primarily affect the basal ganglia, thalamus, and cortex [41]. We reported bilateral cerebral white matter and cerebellum abnormalities as the most common brain MRI finding.

To date, only 8 case reports evaluating 11 patients have been published reporting delayed-onset methadone-induced leukoencephalopathy [6, 10, 16–20], summarized in Table 5. The most frequent imaging findings in case reports of patients with DOL is bilateral cerebral white matter T2 and FLAIR hyperintensity [6, 8, 9, 16, 18, 20] followed by corpus callosum [9, 16] and globus pallidus [8] involvement. This is in keeping with our observation of bilateral cerebral white matter hyperintensity. However, the findings in DOL group are not generalizable, as there were only two cases in this group, who also lacked imaging in their acute phase for comparison with the DOL phase imaging. Furthermore, during examination of DWI and ADC, no restriction was found in either case. Four patients have been described with restriction in DWI scans, although a correlation with ADC was not reported in them [9, 17, 18, 20]. It is possible that the restrictions observed in these patients is related to T2 shine through, as this phenomenon has also been observed in our patients.

**Table 5:** Summary of published works on DOL

Author(s) year	Age/ Sex	Toxic agent	Time to relapse after initial intoxication	Clinical presentation in relapse phase	First imaging findings	Delayed phase MRI findings	DWI and ADC sequences findings	Prognosis
Ljungar B. et al. 2014 [17]	34 yr, M	Methadone	33 days	Physical, psychological and cognitive deterioration	Cerebral white matter T2 hyperintensity	Cystic changes in bilateral cerebral white matter	Hyperintensity in DWI without ADC confirmation	Recovery
Mittal M. et al. 2010 [6]	38 yr, M	Methadone, benzodiazepine	3 weeks	Physical, psychological and behavioral manifestations	No imaging	Cerebral white matter T2 hyperintensity	No restriction	Partial recovery
Arciniegas 2004 [16]	24 yr, M	Methadone, diazepam	Not mentioned	Apathy, disorientation	No imaging	Cerebral white matter and corpus callosum T2 hyperintensity	Not mentioned	Partial recovery
Torralba A- Moron 2016 [8]	42 yr, M	Methadone, alcohol, benzodiazepine	13 days	Myoclonus, fluctuated consciousness,	Normal CT scan	Cerebral white matter and globous pallidus T2 hyperintensity	Not mentioned	Lack of attention and dysexecutive and amnestic abnormalities persisted
	43 yr, M	Methadone	Not mentioned	low level of consciousness and a bradypnoea	Not mentioned	Cerebral white matter and globous pallidus T2 hyperintensity	Not mentioned	death
Zanin A. 2010 [19]	30mo, F	Methadone	19 days	Agitation, slurred speech, abnormal movement	Normal	Tempromesial, Substantia Nigra and basal ganglia	No restriction	Recovery after 2 month
Andrew Meyer M. 2013 [20]	43 yr, F	Methadone, diazepam	3 weeks	Forgetful& confused, social withdrawal, lack of hygiene	No imaging	Cerebral white matter T2 hyperintensity	Hyperintensity in DWI without ADC confirmation	Recovery
Carroll I. 2012 [9]	43 yr, F	Alprazolam, methadone	33 days	Apathy, inappropriate behavior	No imaging	Cerebral white matter and corpus callosum T2 hyperintensity	Corpus Collosum hyperintensity in DWI without ADC confirmation	Full recovery after 6 month
Shprecher D. 2008 [18]	39 yr, F	Methadone, cocaine	4 weeks	disorientation	No imaging	Cerebral white matter T2 hyperintensity	Hyperintensity in DWI without ADC confirmation	Partial recovery
	58 yr, F	methadone	21 days	Paranoid and inappropriate behavioral	No imaging	Cerebral white matter T2 hyperintensity	Not mentioned	Partial recovery
	56 yr, F	Methadone, fentanyl, benzodiazepine	15 days	Cognitive deterioration	Not mentioned	Cerebral white matter T2 hyperintensity	Hyperintensity in DWI without ADC confirmation	Partial recovery

Almost all published case reports to date are in adult patients, except for a single case of 30-month-old infant. There are no previous publications on DOL due to other reasons (strangulation, CO poisoning, benzodiazepine overdose, etc.) in adults younger than 30 years [7]. Since both of our patients were also adults, it is possible that DOL is a phenomenon more common among adult patients. DOL has been previously suggested to be due to hypoxia [6, 16]. However, given that neither of our patients had history of prolonged unconsciousness or respiratory depression, hypoxia as an etiology can be excluded. The lucid intervals of one to 5 weeks have been reported in earlier case reports [7], which was reinforced with our cases.

## Conclusion

Methadone intoxication can result in a spectrum of encephalopathies ranging from AOE to DOL which can be diagnosed using MRI findings. Future studies on larger sample sizes are required to elucidate this association with its possible imaging findings. Our study is the first to demonstrate that MRI changes due to methadone intoxication can parallel those observed in PRES in both adults and children. Given that both heroin and morphine have been previously reported to present with changes suggestive of PRES, it is reasonable to extrapolate this to be an opioid class effect. In DOL, bilateral T2 and FLAIR white matter hyperintensity was the common finding. Therefore, in patients with a recent history of methadone intoxication who represent with relapsing neurological symptoms, DOL needs to be considered.

## Data Availability

All data generated or analysed during this study are included in this published article.

## Abbreviations

ADC: Apparent deficient coefficient
AOE: Acute-onset encephalopathy
CNS: Central nervous system
CO: Carbon monoxide
CT: Computed tomography
DOL: Delayed-onset leukoencephalopathy
DWI: Diffusion weighted imaging
FLAIR: Fluid-attenuated inversion recovery
LOC: Loss of consciousness
MRI: Magnetic resonance imaging
PRES: Posterior reversible encephalopathy syndrome
